# Assessing the geographic range of classical swine fever vaccinations by spatiotemporal modelling in Japan

**DOI:** 10.1111/tbed.14171

**Published:** 2021-06-11

**Authors:** Yichi Yang, Hiroshi Nishiura

**Affiliations:** ^1^ Graduate School of Medicine Hokkaido University Kitaku Hokkaido Japan; ^2^ Kyoto University School of Public Health, Yoshidakonoecho Sakyoku Kyoto Japan

**Keywords:** classical swine fever, diffusion, mathematical model, pig, spatial transmission, wild boar

## Abstract

A classical swine fever (CSF) epidemic has been ongoing in Japan since September 2018. The outbreak started in Gifu Prefecture and involved 21 prefectures by the end of October 2020, posing a serious threat to pork industries. The present study was conducted to capture the spatiotemporal dynamics of CSF in Japan and assess the geographic range of the CSF vaccination on pig farms. First infection dates were collected for wild boars and on swine farms by prefecture. A simple statistical model was used to describe the spatiotemporal dynamics of CSF, describing the infection risk in wild boars and the subsequent transmission hazards to swine farms for 47 prefectures. Because the spatial transmission mechanisms and wild boar population dynamics involved substantial uncertainties, 16 models were applied to the empirical data. Estimated hazard parameters were used to predict the risk of infection on swine farms by 15 December 2020 to explicitly evaluate the governmental recommendation for vaccinations on pig farms by prefecture in light of the predicted infection risk in domestic pigs. The best‐fit model for the wild boars indicated that transmission occurred via neighbouring prefectures and involved seasonality. The estimated conditional hazard was 0.008 (95% confidence interval [CI]: 0.001–0.014) per day for infections transmitted from wild boars to swine farms, and the median time from wild boar infection to swine farm infection was 129.4 days (95% CI: 69.5–935.0). Our prediction indicated that prefectures connected by land to those with wild boar infections had a higher risk of infection on swine farms. CSF transmission in Japan likely progressed diffusively via wild boar movement, and tracking wild boar infections may help determine the risk of infection on swine farms. Our risk map highlights the importance of deciding vaccination policies according to predicted risk.

## 1 INTRODUCTION

Classical swine fever (CSF) is a contagious veterinary disease caused by the CSF virus. Once swine are infected, clinical signs appear within 7–10 days, and the clinical spectrum varies from asymptomatic or mild to severe disease with severe diarrhoea and dullness (World Organisation for Animal Health, 2020). Although CSF epidemic activity had been relatively limited in the 20 years prior (Neumann & Hall, 2019), concern over this disease has increased in Japan since its reemergence in September 2018 (Isoda et al., 2020; Ito et al., 2019). As of 27 October 2020, 59 CSF cases have been reported from swine farms in nine prefectures in Japan, resulting in the slaughter of 171,016 domestic pigs (Online Supplementary Figure S1). The spatial spread is thought to have been facilitated by wild boars, and 21 prefectures have reported CSF‐positive cases (Ministry of Agriculture, Forestry and Fisheries, 2020). Consequently, the Japanese government had Japan removed from the list of CSF‐free countries (World Organisation for Animal Health, 2020) and decided to protect swine farms by vaccinating domestic pigs starting in October 2019. As a consequence of vaccinating domestic pigs and loss of CSF‐free status by the OIE (World Organization for Animal Health), pork exportation to the CSF‐free countries was cancelled, thus financially damaging the pork industries.

The current epidemic has affected the elimination plan as well as CSF control in Japan. The Japanese government recommended pig farm vaccinations for 27 prefectures as of 27 October 2020, including all prefectures with confirmed CSF case(s) and their neighbouring prefectures (Tochigi, Chiba, Osaka, Hyogo, Wakayama, Yamagata and Miyagi). Moreover, the government distributed oral vaccine‐containing foods for the wild boar population to extend the control efforts into wild boar habitats. The notification pace has slowed, and only one domestic pig case was confirmed in Gunma Prefecture between 13 March 2020 and 27 October 2020 (Ministry of Agriculture, Forestry and Fisheries of Japan, 2020). Yamagata later experienced a pig farm infection on 25 December 2020 and a wild boar infection on 27 December 2020. However, monitoring the infection status in wild boars is extremely difficult. Although wild boars can potentially carry the CSF virus and spread it to distant swine farms housing susceptible pigs, it is very difficult to immobilise wild boar populations across prefectural borders.

Epidemiological studies have been conducted and published to better understand the CSF transmission dynamics in Japan. Hayama et al. (2020) used high‐resolution spatial data and a geographic information system to estimate the risk of wild boars infecting swine farms inside Gifu Prefecture. Similarly, Ito et al. (2019) applied a spatiotemporal model to compute the risk of infection in the swine population within Gifu Prefecture over space and examined possible control of the wild boars. Isoda et al. (2020) demonstrated that wild boars frequently transmitted CSF to swine farms. Transmission dynamics have also been explored for African swine fever, and studies have been conducted on host species of CSF (Andrey et al., 2020; Barongo et al., 2015; Gulenkin et al., 2011; Halasa et al., 2016; Iglesias et al., 2016, 2017, 2018; Korennoy et al., 2014; Kukielka et al., 2016; Lu et al., 2019; O'Neill et al., 2020; Oganesyan et al., 2013; Pautienius et al., 2018). Despite these epidemiological modelling efforts, the future prospects of spatiotemporal dynamics to account for CSF transmission across Japan remains uncertain. Quantifying the infection risk to swine in each prefecture over time will enable better vaccination policies. The present study was conducted to devise a simple mathematical model of CSF for all of Japan, calculating the infection risk in both the swine and wild boar populations for each prefecture. Using the predicted risk, we evaluated the policy advice of the vaccination campaign.

## 2 METHODS

### 2.1 Epidemiological data

We collected datasets on wild boar and swine infections from the Ministry of Agriculture, Forestry and Fisheries (Ministry of Agriculture, Forestry and Fisheries, 2021). Infected wild boars or swine were confirmed via reverse transcriptase polymerase chain reaction (RT‐PCR). We gathered data from reports of infected wild boars and swine farms by prefecture and analysed the data available through December 2019.

In addition to the epidemiological data, we obtained wild boar population estimates from two data sources: reports of damage to agricultural products (e.g., vegetables) (Ministry of Agriculture, Forestry and Fisheries, 2017) and reported counts of captured wild boar (Tokyo Agricultural Promotion Bureau, 2018, 2020; Kanagawa Prefectural Government, 2018; Official Statistics of Japan, 2018).

In the following analyses, we used the first date on which a wild boar infection was confirmed and the first date on which a swine farm infection was confirmed. Because swine farm infections were predicted based on wild boar infections, we defined the swine farm infection as the first farm infection that was likely to have acquired the infection from wild boar. That is, we excluded swine farm infection data that were demonstrated to have been caused by transport‐related infections. When the first date of the swine farm infection preceded that of the wild boar infection (seen in Gifu and Saitama), we regarded this as having been due to a delayed diagnosis in the wild boar and excluded such cases from the analysis.

### 2.2 Spatiotemporal modelling

We modelled CSF spatiotemporal dynamics in both wild boars and swine. We first modelled the spatial spread of the infection in wild boars and subsequently used the estimated results to quantify the risk of infection on swine farms. To model the infection hazard in wild boars, we used the relative susceptibility of wild boars:

$$x\;=\beta_{1}\;y/L_{w}+\beta_{2}n_{s}/L_{s},$$

where *x* measures the relative susceptibility of the wild boars and *y* is assumed proportional to the wild boar population size (either by using the agricultural damage data or the captured wild boar counts). Both measurements were used independently; *n_s_* is the domestic pig population size (Official Statistics of Japan, 2018); $L_{s}$ denotes the prefectural area; and $L_{w}$ denotes either the prefectural area or the forest area inside a prefecture (Forest Coverage/Planted Forest Coverage as of March 31, 2017; Forestry Agency, 2017). We also accounted for seasonality as follows:

$$s\left(t\right)\propto \cos\left(\frac{2\pi t}{365}-\varepsilon \right)+1=\cos\left(\frac{2\pi \left(t-365\varepsilon /2\pi \right)}{365}\right)+1,$$

where *ε* adjusts for the timing of seasonal variations in time, and the unit of time *t* is days, starting from 13 September 2019, the date when the first wild boar case was reported in Japan. To model the infection hazard in wild boars, we used two approaches: the gravity model and the neighbourhood model. The gravity model uses the Euclid distance to determine the distance‐dependent decay in the transmission risk:

$$a_{ij}\left(t\right)=\;s\left(t\right)x_{i}d_{ij}^{-2},$$

where *a_ij_* is the hazard rate of transmission from prefecture *j* to prefecture *i*, *s*(*t*) and *x_i_* are as described above, and *d_ij_* is the Euclidean distance between prefectures *j* and *i* as measured by the location of the prefectural headquarter office. Alternatively, the neighbourhood model is formulated as

$$a_{ij}\left(t\right)=\;s\left(t\right)x_{i}\theta_{ij\;},$$

where *θ* is the adjacency matrix (*θ*, {0, 1}), that is, if a land connection exists between prefectures *i* and *j*, and if *j* was infected, $\theta_{ij}=\;1$, otherwise, $\theta_{ij}=\;0$.

Using either hazard function, the daily infection risk from wild boars in prefecture *i* on day *t* is

$$R_{i}\left(t\right)\;=\;1-\prod_{j}\exp\left(-a_{ij}\left(t\right)\right).$$

To estimate the parameters governing the hazard function of wild boar infections in prefecture *i*, we defined the event time as the date of the first report in the *m*th prefecture as *t_m_*. For example, the date of the first report in Gifu (the first affected prefecture) was *t*_0_ = 0 (13 September 2018). The time in Aichi (the second affected prefecture) was *t*_1_ = 21 December 2018 to 13 September 2018 (21 December 2018 is the date that the first wild boar case was reported in Aichi) and continuing through the last *t_m_*. The empirical observation was censored on 15 December 2019:

$$\begin{array}{ccc} \frac{Gifu}{t_{0}} & = & \;>\;\frac{Aichi}{t_{1}}=\;>\;\frac{Mie}{t_{2}}=\;>\;\frac{Fukui}{t_{3}}=\;>\;\frac{Nagano}{t_{4}}\\ & = & \;>\;\frac{Toyama}{t_{5}}=\;>\;\frac{Ishikawa}{t_{6}}=\;>\frac{Shiga}{t_{7}} \end{array}$$

$$\begin{array}{ccc} \; & = & \;>\;\frac{Saitama}{t_{8}}=\;>\;\frac{Gunma}{t_{9}}=\;>\;\frac{Shizuoka}{t_{10}}=\;>\;\frac{Yamanashi}{t_{11}}\\ & = & \;>\frac{Current}{t_{12}} \end{array}$$

*t_w_* was the event time of prefecture *i*. Given the previous event on day *t_w_*_–1_, the conditional likelihood of observing infected wild boars in prefecture *i* is the product of the risk of infection in prefecture *i* on *t_w_* and the escape probability of all other prefectures *j*. Thus, the log‐likelihood is

$$\;l_{w}=\sum_{m=t_{w-1}+1}^{t_{w}-1}\log\left(1-R_{i}\left(m\right)\right)\;+\log R_{i}\left(t_{w}\right)+\sum_{j}\sum_{n\;=t_{w-1}\;+1}^{t_{w}}\log\left(1-R_{j}\left(n\right)\right).$$

The total log‐likelihood is then calculated as

$$l\;=\sum_{w}l_{w}\;.$$

### 2.3 Modelling the hazard for domestic pigs

Subsequently, we modelled the risk of infection in domestic pigs. As was applied to wild boars, the swine population risk was also based on the hazard function. Specifically, we modelled the daily transmission risk in pigs as

R=1−exp(−δt),

where δ represents the hazard for transmission from wild boars to swine farms and was treated as an unknown parameter. That is, we counted the risk from the date of infection in the wild boars. If the first wild boar case was reported in prefecture *i* on day *t_si_*, then the first domestic pig infection was reported on day *t_ei_*. Among prefectures with infected swine farms, the likelihood used to estimate the hazard rate δ was

$$\;L_{1}=\prod_{i}\left(1-\exp\left(-\delta \right)\right)\exp\left(-\delta \left(t_{ei}-t_{si}-1\right)\right)\;.$$

If *t_n_* is the latest observation date, using prefectures *j* with no reports of infected swine farms yields

$$\;L_{2}=\prod_{j}\exp\left(-\delta \left(t_{ei}-t_{si}\right)\right)\;.$$

The total likelihood is $L_{1}L_{2}$. Maximum likelihood estimation was implemented to optimise the model and obtain parameter estimates.

### 2.4 Estimation scenarios and real‐time forecasting

We used the above likelihood functions to estimate unknown parameters. Day zero was the date on which the first wild boar infection was reported from Gifu Prefecture. This yielded 16 scenarios for comparison, which arose from four dichotomous combinations: (i) whether we adopted a gravity model or neighbourhood‐transmission model to model the infection risk in wild boars, (ii) whether we accounted for seasonality *s*(*t*) in the infection hazards of wild boars, (iii) whether we used the agricultural damage data or the captured wild boar counts to approximate the prefectural variations in the wild boar population size, *x*, and (iv) whether we used the prefecture area or the forest area to calculate the wild boar density.

For each scenario, we predicted the infection by 15 December 2020. We stochastically modelled the risk of prefecture *i* per day using $1-\exp(-a_{ij})$. The predicted risk of infection was compared against the presence of a vaccination program in the swine population by prefecture.

A receiver operating characteristic (ROC) curve was drawn to compare the model estimate with the observed value to compare the model performances. To identify the cut‐off value for the infection risk either in wild boars or on pig farms, we used Youden's J statistic (Youden index). The ROC is a graphic tool that sets the false positive rate as the *x* axis and the true positive rate as the y axis to evaluate the model's performance and choose the best cut‐off value (Hoo et al., 2017). The Youden index was defined as sensitivity + specificity − 1, and the point at which the maximum Youden index was obtained was used as the optimal cut‐off point (Ruopp et al., 2008). The predicted infection risk on 15 December 2019 in wild boars or on pig farms was then computed for each prefecture, and prefectures that were predicted to have infections in wild boars or on pig farms were considered ‘high‐risk’ prefectures that may require vaccinations for the wild boars or pig farms.

### 2.5 Data sharing statement

The first reporting dates for the wild boars and swine farms by prefecture are presented in the online supporting material (Supplementary Table S1).

## 3 RESULTS

**FIGURE 1:**
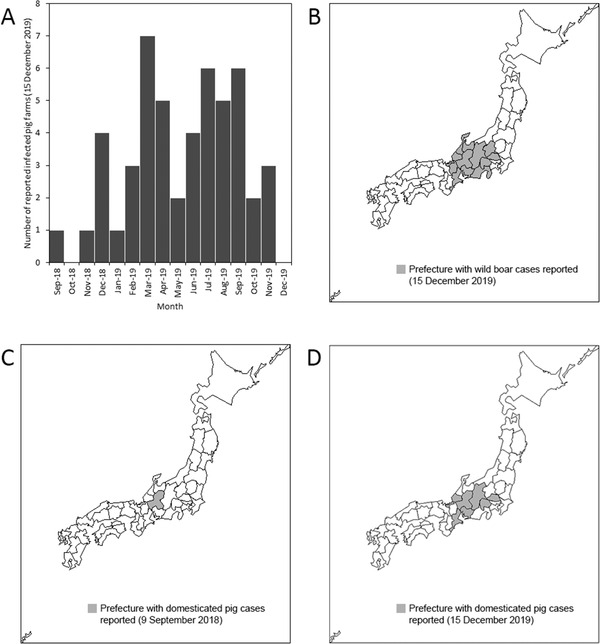
Epidemiology of classical swine fever in Japan as of 15 December 2019. (a) Number of newly reported infected swine farms. The vertical axis shows the number of farms; the horizontal axis shows the reporting month. (b) Geographic distribution of prefectures with reported wild boar infections. As of 15 December 2019, twelve prefectures experienced infections (shown in grey). (c) First reported swine farm in Gifu Prefecture, September 2018. (d) Geographic distribution of prefectures with reported swine farm infections. Seven prefectures reported swine farm outbreaks, 15 December 2019

Figure 1 shows the epidemiological dynamics over time and space. Fifty swine farms reported confirmed infections by 15 December 2019 (Figure 1A), and 12 prefectures reported wild boar infections (Figure 1B). Gifu Prefecture reported the first swine farm infection (Figure 1C), and seven prefectures reported having infected swine farms by 15 December 2019 (Figure 1D). Table 1 summarises and compares the model performances. Among all 16 possible model combinations, scenario 5, the neighbourhood‐transmission model with seasonality using agricultural damage data and prefectural area data to mirror the wild boar population dynamics, yielded the lowest Akaike information criterion (AIC) value (147.0). Model 13, which used identical model assumptions while using forest area to calculate the wild boar density, was the next best‐fit model (AIC = 147.3). These results favouring neighbourhood transmission indicated that the transmission occurred diffusively across geographic space (e.g., via wondering behaviour to neighbouring prefectures) and that seasonal variations in transmissibility or movement of infected wild boars played a role in the geographic spread. The seasonality parameter was estimated at ε=5.865, indicating that seasonal forcing peaked at *t* = 341 days (where *t*_0_ is 13 September 2018). This finding suggests that CSF transmission among wild boars intensifies in the summer. This may because of the warm climate in summer activate the movement of wild boar. Wild boars born in the last year getting independence may be another considerable reason.

**TABLE 1:** Parameter estimates of various hazard models for the classical swine fever outbreak in Japan

Combination scenarios	Coefficient for wild boar population size (*β*_1_)	Coefficient for swine population density (*β*_2_)	Location parameter for the seasonality (ε)	Akaike Information Criterion
1 (neighbour + agriculture damage + no seasonality + province area)	16.24	0.04	‐	150.1
2 (neighbour + capture data + no seasonality + province area)	9.28	0.07	‐	154.0
3 (gravity + agriculture damage + no seasonality + province area)	1.90	0.01	‐	173.2
4 (gravity + capture data + no seasonality + province area)	0.68	0.02	‐	178.1
5 (neighbour + agriculture damage + with seasonality + province area)	14.47	0.03	5.86	147.0
6 (neighbour + capture data + with seasonality + province area)	8.47	0.06	5.68	150.7
7 (gravity + agriculture damage + with seasonality + province area)	2.17	0.01	5.47	169.4
8 (gravity + capture data + with seasonality + province area)	0.75	0.02	5.41	174.2
9 (neighbour + agriculture damage + no seasonality + forest area)	10.88	0.03	‐	150.4
10 (neighbour + capture data + no seasonality + forest area)	4.65	0.08	‐	155.3
11 (gravity + agriculture damage + no seasonality + forest area)	1.26	0.01	‐	173.9
12 (gravity + capture data + no seasonality + forest area)	0.25	0.02	‐	179.1
13 (neighbour + agriculture damage + with seasonality + forest area)	9.45	0.02	5.81	147.3
14 (neighbour + capture data + with seasonality + forest area)	4.78	0.07	5.60	151.6
15 (gravity + agriculture damage + with seasonality + forest area)	1.24	0.01	5.46	170.3
16 (gravity + capture data + with seasonality + forest area)	0.29	0.02	5.41	175.2

Note: Parameters *β*_1_ and *β*_2_ are the coefficients for factoring the population impact of the wild boar and swine density in each prefecture in the hazard function. Parameter ε helped identify the location for the seasonality. Neighbour and gravity refer to the neighbourhood transmission and gravity (distant‐dependent) models, respectively, for the wild boar infection hazard.

**FIGURE 2:**
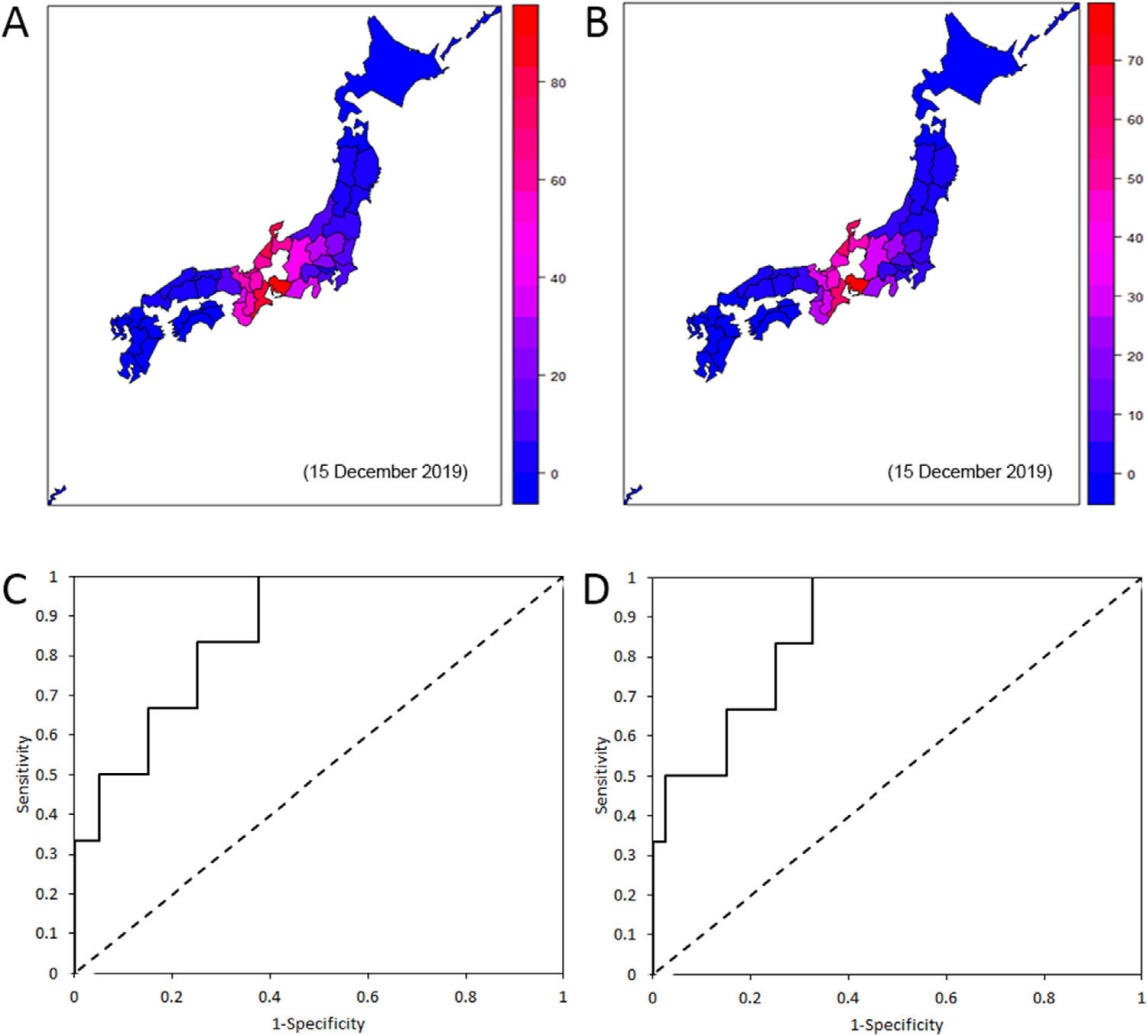
Predicted risk of infection in wild boars and on swine farms. Panels a and c: wild boar; Panels b and d: swine farms. Panels a and b show the risk of wild boar and swine farm infections by December 2019 (15 months after the first outbreak in Gifu, September 2018, using the best‐fit model, i.e. neighbourhood transmission for wild boar + agricultural damage data for wild boar population + seasonality in the infection hazard + province area for wild boar density calculation). The prefecture of origin, Gifu Prefecture, is shown in white. The colour of each prefecture represents the probability of CSF cases being reported. Panels c and d show the predictive performance by prefecture. The receiver operating characteristic curve shows the estimated cut‐off value for the risk of infection. The optimal cut‐off value for infections on swine farms was estimated at 0.15

Figure 2A shows the predicted geographic distribution of the infection risk from wild boars by 15 December 2019, using model 5 (i.e., the best‐fit model) with the minimum AIC. CSF‐free prefectures that geographically neighboured infected prefectures (Ibaraki, Tochigi and Chiba) yielded higher infection risks than did other distant prefectures (see Online Supplementary Figure S2 for prefecture map). Figure 2B shows the risk map for swine farm infections using the same algorithm as used in Figure 2A. The hazard rate δ that governs the gap time from wild boar infection to swine farm infection was estimated as 0.008 (95% confidence interval [CI]: 0.001–0.014) per day. Figures 2C and 2D show the respective ROC curves that compare the observed and predicted conditions of the wild boar and swine farm infections on 15 December 2019. The risk of infection on swine farms by 15 December 2019 was obtained using the best‐fit model (Table 2), and the area under the curve (AUC) was estimated at 86.3% for the wild boars and 87.5% for the swine farms. Model 13, the second best‐fit model, estimated the maximum AUC for wild boar infections at 85.0%. Similarly, model 1 (neighbourhood transmission without seasonality using agricultural damage data) estimated the maximum AUC at 87.1%. The optimal cut‐off value for swine farm infections using model 5 (the best‐fit model) was 0.153, with a Youden index of 0.675.

**TABLE 2:** Comparison between model‐predicted risk and actual infection and interventions (as of 27 October 2020)

Prefectures	Presence of wild boar infection	Presence of swine farm infection	Vaccination implemented for the swine farms	Swine farms vaccination recommended prefectural(20 December 2019)	Predicted risk of swine farm infection from the best fit model (15 December 2019)
Hokkaido	0	0	0	0	0.00
Aomori	0	0	0	0	0.00
Iwate	0	0	0	0	0.00
Miyagi	0	0	1	0	0.03
Akita	0	0	0	0	0.01
Yamagata	0	0	1	0	0.05
Fukushima	1	0	1	0	0.10
Ibaraki	1	0	1	1	0.19
Tochigi	0	0	1	1	0.22
Gunma	1	1	1	1	0.36
Saitama	1	1	1	1	0.23
Chiba	0	0	1	1	0.15
Tokyo	1	0	1	1	0.03
Kanagawa	1	0	1	1	0.16
Niigata	1	0	1	1	0.19
Toyama	1	0	1	1	0.69
Ishikawa	1	0	1	1	0.80
Fukui	1	1	1	1	0.67
Yamanashi*	1	1	1	1	0.15
Nagano*	1	1	1	1	0.53
Gifu	1	1	1	1	1.00
Shizuoka	1	0	1	1	0.44
Aichi	1	1	1	1	0.93
Mie	1	1	1	1	0.81
Shiga*	1	0	1	1	0.64
Kyoto	1	0	1	1	0.58
Osaka*	0	0	1	0	0.35
Hyogo	0	0	1	0	0.18
Nara	1	0	1	1	0.59
Wakayama	0	0	1	0	0.52
Tottori	0	0	0	0	0.04
Shimane	0	0	0	0	0.01
Okayama	0	0	0	0	0.03
Hiroshima	0	0	0	0	0.02
Yamaguchi	0	0	0	0	0.00
Tokushima	0	0	0	0	0.00
Kagawa	0	0	0	0	0.00
Ehime	0	0	0	0	0.00
Kochi	0	0	0	0	0.00
Fukuoka	0	0	0	0	0.00
Saga	0	0	0	0	0.00
Nagasaki	0	0	0	0	0.00
Kumamoto	0	0	0	0	0.00
Oita	0	0	0	0	0.00
Miyazaki	0	0	0	0	0.00
Kagoshima	0	0	0	0	0.00
Okinawa	0	1	1	0	0.00

Note: 1: present; 0: absent.

*The swine farm infections in Osaka Shiga and Nagano on 2/6/2019 were transport‐related infections from Aichi. The disease was controlled before the virus spread; thus, Osaka is still considered a CSF‐free prefecture, and Shiga is considered to have no pig farm cases in our geographic model. Similarly, a transport‐related infection in Yamanashi on 13 September 2020 was considered to have come from a swine farm in Saitama. Additional cases in wild boars were diagnosed on 29 October 2020 in Osaka and 30 October 2020 in Wakayama. These two cases were not reflected because our latest date of observation was 27 October 2020.

**FIGURE 3:**
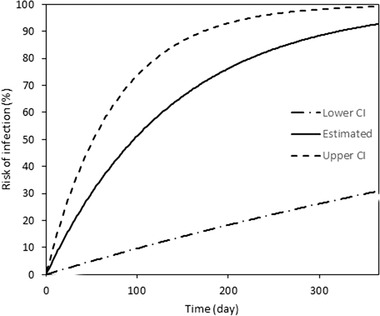
A constant hazard and its confidence interval for pig farms becoming infected after a wild boar case is reported. The vertical axis presents the infection risk; the horizontal axis shows the time. The infection risk increases if no intervention occurs. The break lines show the upper and lower confidence intervals separately

Figure 3 shows the risk of infection from wild boars to swine, given the infection in wild boars in an identical prefecture as a function of the time since the wild boar infection was identified. The median time from wild boar infection to swine farm infection was derived as an inverse of the estimated hazard rate (δ), calculated as 129.4 days (95% CI: 69.5–935.0), indicating that the upper bound limit involves substantial uncertainty.

**FIGURE 4:**
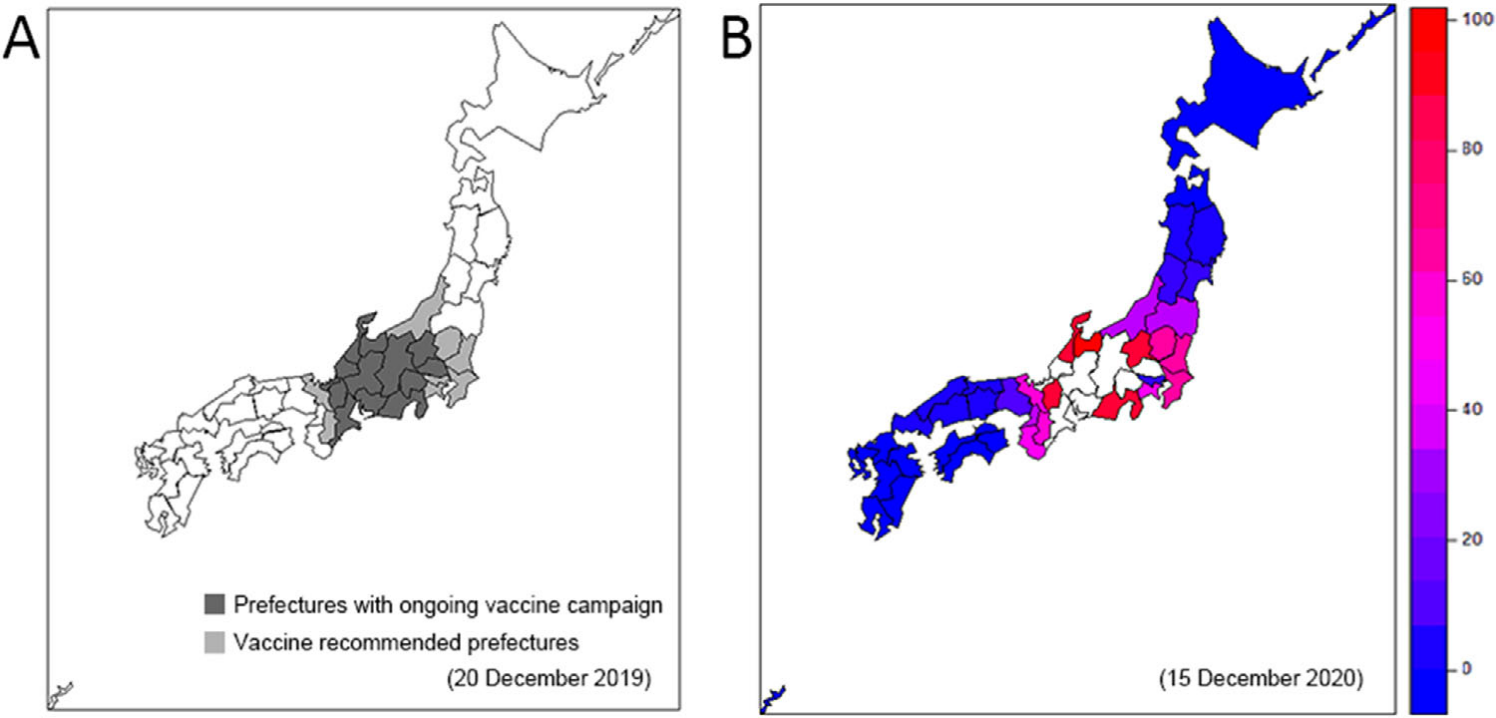
Vaccine conditions for 20 Dec 2019 and predicted risk map for 15 Dec 2020. (a) Vaccine campaign conditions by 20 Dec 2019. Prefectures with ongoing vaccine campaigns for domesticated pigs are in dark grey; vaccination recommended prefectures are in light grey. (b) Predicted risk map for domesticated pig farms 1 year later (15 Dec 2020). White prefectures indicate areas that had reported pig farm cases by 15 December 2019. Of note, eight other prefectures (Tochigi, Ibaraki, Chiba, Tokyo, Kanagawa, Niigata, Kyoto and Nara) were additionally designated as prefectures recommended for pig farm vaccinations on 20 December 2019

Figure 4A shows the prefectures with swine farm vaccinations as of 20 December 2019. Chiba, Kanagawa, Ibaraki, Tochigi, Nara, Niigata, Tokyo and Kyoto were also officially recommended swine farm vaccinations by the Japanese government. Compared with the spatial distribution of the vaccinations, Figure 4B shows the predicted risk of infection on swine farms by 15 December 2020. High‐risk areas are mostly overlaid with prefectures containing vaccinated farms (Gunma, Toyama, Ishikawa, Shizuoka and Shiga) and a part of vaccine recommended prefectures(Chiba, Kanagawa, Ibaraki, Tochigi, Nara, Niigata and Kyoto) but also included Fukushima, Wakayama and Osaka as having a high risk for swine farm infection.

Online Supplementary Table S1 shows the empirical data as of 27 October 2020. Seven additional prefectures (Ibaraki, Fukushima, Tokyo, Kanagawa, Niigata, Kyoto and Nara) reported wild boar infections, and all were predicted to be at risk in our model. Nevertheless, only one additional swine farm case was reported in Gunma (26 September 2020), and a distant prefecture infection was reported in Okinawa (8 January 2020), the southernmost prefecture. Online Supplementary Figure S1 shows the updated epidemic curve. In addition to Okinawa in January 2020, seven other swine farms became newly infected.

## 4 DISCUSSION

To appropriately quantify the spatiotemporal model of CSF transmission in Japan, we analysed both wild boar and swine datasets using a parsimonious approach and compared all possible model combinations. Sixteen combinations were compared, and the best‐fit model indicated that the neighbourhood‐transmission model would capture the reality better than would the distance‐based model, and the transmission pattern may involve seasonality. Using agricultural damage data for wild boar populations yielded slightly better results than did using ecological observation of the capture data. The predictive performance of the best‐fit model was satisfactory; the AUC values for wild boars and swine were 86.3% and 87.5%, respectively. The swine infection risk was computed by simulating the epidemic by December 2020 using the estimated parameters. Prefectures with ongoing vaccinations agreed well with those at high risk, but our model also highlighted additional prefectures, including Fukushima, Niigata, Wakayama and Osaka, as high‐risk prefectures.

The most important take‐home message from the present study is that the CSF transmission risk on swine farms in Japan is characterised by wild boar infections in the same or neighbouring prefecture(s). The risk from wild boar‐induced transmission, rather than from swine‐induced transmission, regulates the ongoing transmission risk to swine. The immediate policy suggestion is to control wild boar‐induced transmission. If wild boar mobility cannot be managed manually, prefectures with infected wild boars and their neighbouring prefectures should receive oral vaccine supplies for the wild boars. Moreover, to prevent massive economic damage to swine farms, these prefectures should also receive swine vaccinations. Vaccinating swine in prefectures with wild boar infections and in prefectures directly connected to those with wild boar infections would likely be the key to success.

What is the previously unknown critical aspect for controlling CSF in Japan? Transmission across neighbouring prefectures via wild boar movement is an important feature of this epidemic. If a prefecture is geographically distant and has no wild boars moving directly from infected prefectures, the risk of infection to both wild boars and swine farms in that prefecture can be minimised. Because the ongoing epidemic is spatially diffused directly from Gifu to other neighbouring prefectures, protecting risk‐facing infection‐free prefectures in the western, eastern and northeastern regions (Kansai, Kanto and Tohoku) is the key to protecting other distant prefectures. Thus, ‘ring vaccinations’ would be crucial macroscopically for successfully controlling this epidemic throughout Japan. Importantly, the oral vaccinations for wild boars that started in March 2019 have not fully prevented the spatial spread across Japan, and the lack of mobility control among wild animals has complicated controlling the epidemic.

In our study, we used a spatiotemporal model to address the infection risk on a prefectural scale. Analyzing a finer spatial data scale would be quantitatively more useful. Given the higher resolution data, more targeted approaches, such as targeting a certain spatial unit of swine farms or focusing on specific wild boar habitats, could be considered. Seroepidemiological surveys could also be considered for future investigations. Because vaccine campaigns have been implemented for wild boars and swine, antibodies should be detected in these animals regardless of whether the animals were artificially immunised or naturally infected. Seroepidemiological surveillance may be used to identify spatial hotspots of infection, and local vaccination campaigns could potentially refer to these datasets.

Our study had three limitations. First, wild boar population data were limited. The scale of the agricultural activities in each prefecture could have biased the agricultural damage caused by wild boars. Similarly, the number of wildlife hunters per prefecture could have biased the number of captured wild boar. Second, additional geographic information, such as land use data, was not explored. The wild boar population density was likely highly varied owing to the presence of forests with rivers or ponds and agricultural farms. Third, we assumed that the infection hazard to swine farms was a constant when estimating the time delay from wild boar infection to swine farm infection in the same prefecture. Potential seasonal population dynamics and behaviours would induce variations, and future studies should explore possible alternatives.

Despite these limitations, the present study helped clarify the role of wild boars in propagating CSF across geographic locations and on swine farms. Vaccination campaigns must account for the existence of wild boar infections in the same and neighbouring prefectures.

## ETHICAL STATEMENT

No samples or primary data were collected from animals or humans in the present study; thus, no ethical approval was required.

## CONFLICT OF INTEREST

The authors declare that they have no conflicts of interest.

## Supporting information



supporting information

Figure S2. Japanese prefecture map
