# Supplementary material for: Assessing the geographic range of classical swine fever vaccinations by spatiotemporal modelling in Japan
Source: Transbound Emerg Dis. 2021 Jun 11;69(4):1880–9. doi: 10.1111/tbed.14171 (PMC9546044; doi:10.1111/tbed.14171)
Supplement: Supplementary file 1 — supporting information [file TBED-69-1880-s001.docx]

**Supplementary Table S1. Date on which the first classical swine fever infection event occurred in Japan by prefecture as of 27 October 2020**

| Prefectures | Date of the first wild boar case confirmed | Date of the first domestic pig case confirmed | Ongoing domesticated pig vaccine |
| --- | --- | --- | --- |
| Hokkaido | NA | NA | 0 |
| Aomori | NA | NA | 0 |
| Iwate | NA | NA | 0 |
| Miyagi | NA | NA | 1 |
| Akita | NA | NA | 0 |
| Yamagata | NA | NA | 1 |
| Fukushima | 9/9/2020 | NA | 1 |
| Ibaraki | 6/27/2020 | NA | 1 |
| Tochigi | NA | NA | 1 |
| Gunma | 10/4/2019 | 9/26/2020 | 1 |
| Saitama | 9/24/2019 | ^†^9/13/2019 | 1 |
| Chiba | NA | NA | 1 |
| Tokyo | 7/2/2020 | NA | 1 |
| Kanagawa | 5/11/2020 | NA | 1 |
| Niigata | 4/24/2020 | NA | 1 |
| Toyama | 7/28/2019 | NA | 1 |
| Ishikawa | 8/22/2019 | NA | 1 |
| Fukui | 7/6/2019 | 7/29/2019 | 1 |
| Yamanashi | 10/31/2019 | *11/16/2019 | 1 |
| Nagano | 7/13/2019 | *9/14/2019 | 1 |
| Gifu | 9/13/2018 | ^†^9/9/2018 | 1 |
| Shizuoka | 10/18/2019 | NA | 1 |
| Aichi | 12/21/2018 | 2/6/2019 | 1 |
| Mie | 6/26/2019 | 7/24/2019 | 1 |
| Shiga | 9/18/2019 | *NA | 1 |
| Kyoto | 4/27/2020 | NA | 1 |
| Osaka | NA | *NA | 1 |
| Hyogo | NA | NA | 1 |
| Nara | 10/14/2020 | NA | 1 |
| Wakayama | NA | NA | 1 |
| Tottori | NA | NA | 0 |
| Shimane | NA | NA | 0 |
| Okayama | NA | NA | 0 |
| Hiroshima | NA | NA | 0 |
| Yamaguchi | NA | NA | 0 |
| Tokushima | NA | NA | 0 |
| Kagawa | NA | NA | 0 |
| Ehime | NA | NA | 0 |
| Kochi | NA | NA | 0 |
| Fukuoka | NA | NA | 0 |
| Saga | NA | NA | 0 |
| Nagasaki | NA | NA | 0 |
| Kumamoto | NA | NA | 0 |
| Oita | NA | NA | 0 |
| Miyazaki | NA | NA | 0 |
| Kagoshima | NA | NA | 0 |
| Okinawa | NA | 1/8/2020 | 1 |

*Transport-related swine farm infections that occurred on 6 February 2019 were disregarded in Shiga, Osaka and Nagano because these prefectures had acquired infections from a previously infected farm in Aichi. As of 27 October 2020, Shiga and Osaka have experienced no further swine farm infections and are thus designated as not available (NA). In Nagano, the swine farm epidemic started on 14 September 2019. Similarly, a transport-related infection in Yamanashi on 13 September 2020 was considered to have come from a swine farm in Saitama. ^†^In Gifu, the index prefecture, the swine farm infection occurred before the wild boar infection. This was also the case for Saitama, where the swine became infected on 13 September 2019, and the wild boar became infected on 24 September 2019, likely because the diagnosis was delayed in the wild boar. Additional cases in wild boars were diagnosed on 29 October 2020 in Osaka and 30 October 2020 in Wakayama. These two cases are not included in the table because our latest observation date was 27 October 2020.
