# Supplementary material for: Assessing the geographic range of classical swine fever vaccinations by spatiotemporal modelling in Japan
Source: Transbound Emerg Dis. 2021 Jun 11;69(4):1880–9. doi: 10.1111/tbed.14171 (PMC9546044; doi:10.1111/tbed.14171)

**Supplementary Figure S1. Epidemiology of classical swine fever in Japan as of 27 October 2020.**

**
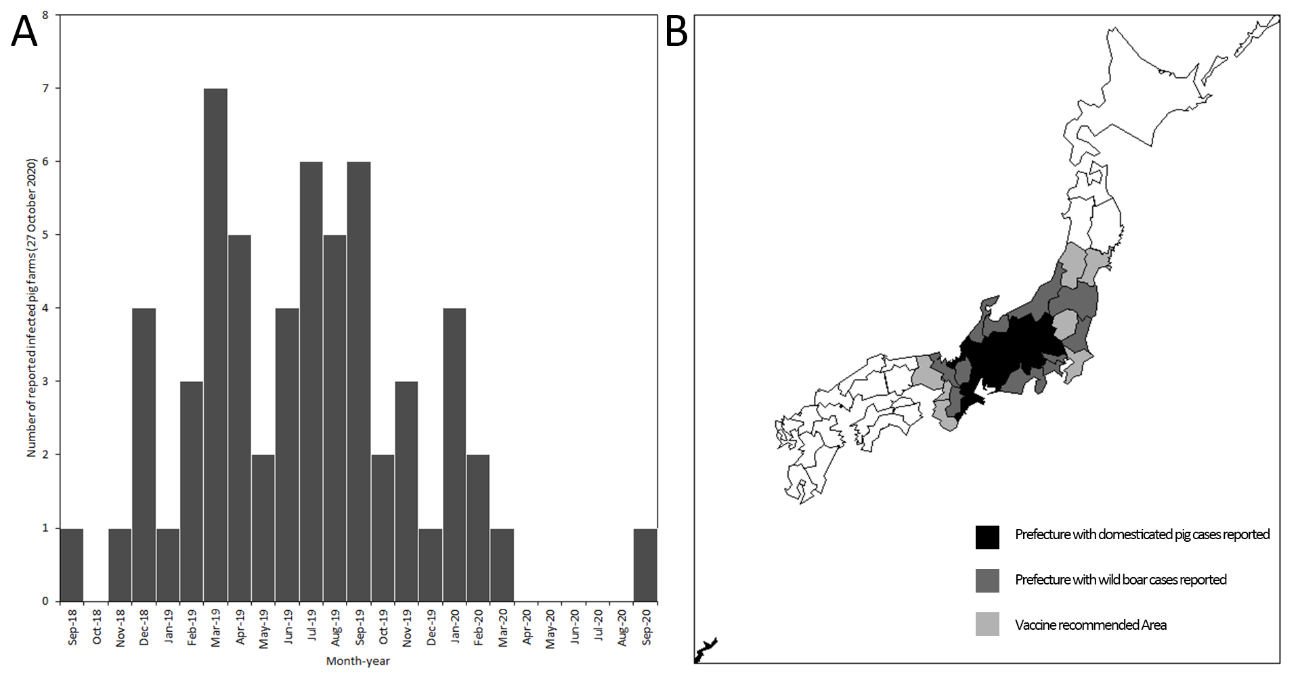
**

**Supplementary Figure S2. The Japanese prefecture map.**


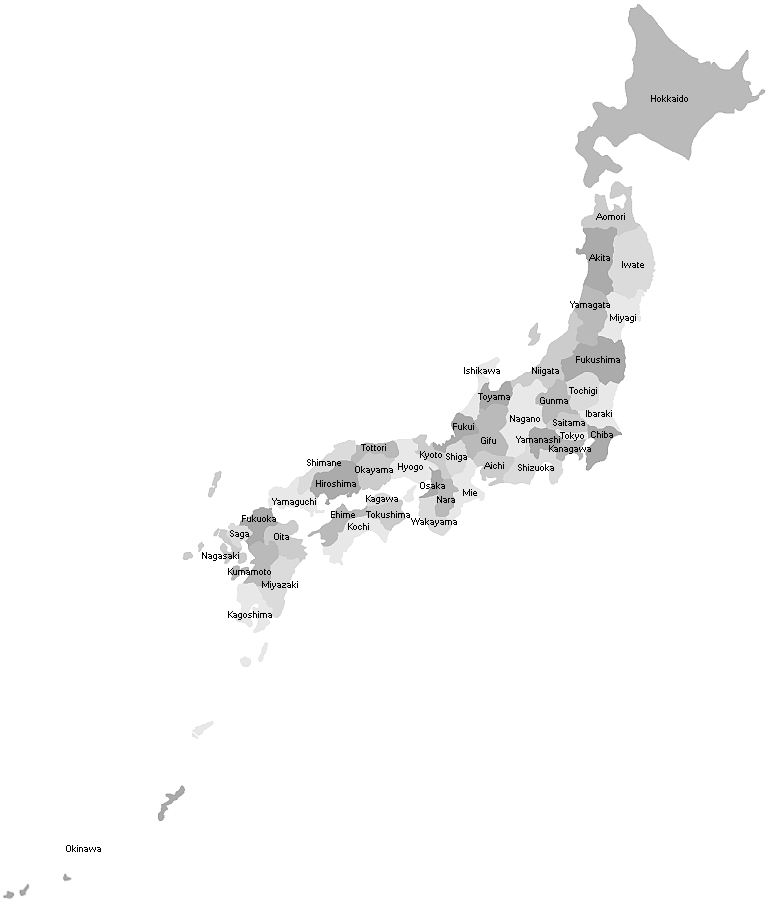

Supplement: Supplementary file 2 — Figure S2. Japanese prefecture map [file TBED-69-1880-s002.docx]
